# Reproductive autonomy and the experience of later-than-desired pregnancy: results from a cross-sectional survey of reproductive-aged women in Uganda

**DOI:** 10.1186/s12978-024-01750-z

**Published:** 2024-02-06

**Authors:** Suzanne O. Bell, Fredrick Makumbi, Isabella Sarria, Simon P. S. Kibira, Linnea A. Zimmerman

**Affiliations:** 1 Department of Population Family and Reproductive Health, Johns Hopkins Bloomberg School of Public Health, 615 N. Wolfe St., Baltimore, MD 21205 USA; 2 Department of Epidemiology and Biostatistics, School of Public Health, College of Health Sciences, Makerere University, Kampala, Uganda; 3 Department of Community Health and Behavioral Sciences, School of Public Health, College of Health Sciences, Makerere University, Kampala, Uganda

**Keywords:** Pregnancy desire, Pregnancy intention, Survey research, Uganda

## Abstract

**Background:**

The focus of reproductive autonomy research has historically been on the experience of unintended pregnancy and use of contraceptive methods. However, this has led to the neglect of a different group of women who suffer from constraints on their reproductive autonomy—women who experience pregnancies later than they desire or who are unable to become pregnant. This study examines the extent of later-than-desired pregnancy among women and evaluates the sociodemographic and reproductive factors associated with this experience in Uganda.

**Methods:**

We use data from the Performance Monitoring for Action Uganda 2022 female survey. We restricted the nationally representative sample of reproductive-aged women to those who were currently pregnant or who had ever given birth (n = 3311). We compared the characteristics of women across fertility intention categories (wanted pregnancy earlier, then, later, or not at all) of their current or most recent birth and used multivariable logistic regression to examine factors independently associated with having a pregnancy later than desired compared to at a desired time.

**Results:**

Overall, 28.3% of women had a later-than-desired pregnancy. Nearly all sociodemographic and reproductive characteristics were associated with the desired pregnancy timing of women’s current or most recent pregnancy. Having higher education [adjusted odds ratio (aOR) 2.41, 95% confidence interval (CI) 1.13–5.13], having sought care for difficulties getting pregnant (aOR 2.12, 95% CI 1.30–3.46), and having less than very good self-rated health (good health aOR 1.74, 95% CI 1.12–2.71; moderate health aOR 1.77, 95% CI 1.09–2.86; very bad health aOR 4.32, 95% CI 1.15–16.26) were all independently significantly associated with increased odds of having a later-than-desired pregnancy. Being nulliparous (aOR 1.98, 95% CI 0.99–3.95) was also borderline significantly associated with having a later-than-desired pregnancy.

**Conclusions:**

Identifying those who have later-than-desired pregnancies is essential if we seek to make progress towards supporting women and couples in achieving their reproductive goals, not just preventing pregnancies. Research on desired pregnancy timing in sub-Saharan Africa should be expanded to capture later-than-desired pregnancies, a population which is invisible in existing data. This work has public health implications due to commonalities in the factors associated with mistimed and unintended pregnancies and their link to poorer health and potentially poorer pregnancy outcomes.

## Background

Though researchers have conceptualized and defined reproductive autonomy in myriad ways [1–3], it is generally considered to be the ability to achieve one’s desired fertility intentions, including the number and timing of children. The focus of reproductive autonomy research has historically been on the experience of unintended pregnancy and use of contraceptive methods [3–6]. This has largely been justified due to the observed associations between unintended pregnancy and multiple negative maternal and newborn health outcomes (e.g., low birthweight, child abuse, maternal morbidity, maternal mental health) [7–10]. This singular focus, however, has led to the neglect of a different group of women who suffer from constraints on their reproductive autonomy—women who experience pregnancies later than they desire or who are unable to become pregnant altogether.

Understanding the experiences and outcomes of women who have a later-than-desired pregnancy is of critical importance given the constellation of associated negative consequences. Research indicates subfecundity and infertility (no pregnancy following 1-year of regular unprotected sex) are associated with increased risk of intimate partner violence, poor mental health outcomes, abandonment and social isolation, and catastrophic spending on treatment [11–15]. Delayed time-to-pregnancy also has potential health implications for the resulting pregnancy as research from high-resource settings suggests these births have an increased risk of low birthweight compared to on-time births [16–20]. Additionally, having a later-than-desired birth positively predicts that the next birth will be mistimed, rather than being on-time [16]. Despite these potential implications for health and well-being, we know relatively little about this population of women.

While there is growing research on infertility, which affects approximately 13% of couples globally [21], significant research gaps exist in understanding even the extent of pregnancies that occur later than desired. In the United States, surveys such as the Pregnancy Risk Assessment Monitoring System (PRAMS) and the National Survey of Family Growth (NSFG) are two of the primary sources of data on pregnancy intentions [22–25]. Women are generally asked to report on whether their most recent pregnancy was wanted at the time it occurred, sooner, later, or not at all. Research using these data generally consider births that were wanted later or not at all as unintended, while those that were wanted then or sooner are generally considered intended (less often births wanted sooner are classified as “mistimed”) [2, 23, 24, 26–29]. This grouping may be justified if the research focus is on pregnancy intention as those desiring a pregnancy sooner are indeed intending to become pregnant, however, this singular “intended” group masks potentially significant heterogeneity in the characteristics, experiences, and outcomes of women who became pregnant later-than-desired [28, 30, 31]. One study in the United States that did separate this group found that 17–18% of women desired the pregnancy sooner [31]. When investigators added a “I wasn’t sure what I wanted” category, women were significantly less likely to respond that they wanted to become pregnant sooner [31]. The addition of a new response affecting the rates among different groups in various ways underscores the heterogeneity of these groups and their experiences, highlighting a need to further understand this population of women and factors influencing their attitudes towards pregnancy timing. An earlier study in the US found that 10% of births were reported as occurring later-than-desired [16]. However, we are aware of no other research exploring this group, including no studies in sub-Saharan Africa.

Recent research indicates that substantial percentages of women in sub-Saharan Africa have had fewer children than desired at the end of their reproductive lifecourse, referred to as unrealized fertility [32–34]. In one study, 64% of women aged 44–48 experienced unrealized fertility in Western and Central Africa and 44% in Eastern and Southern Africa [34]. This is significant given that the consequences of infertility and subfecundity can be profound [35–38]. There remains, however, little known about fertility delays in sub-Saharan Africa, where the bias in the literature is towards understanding and preventing mistimed and unwanted births [39–41]. The Demographic and Health Survey (DHS), the largest data source on fertility in sub-Saharan Africa, does not include answer options for wanting a pregnancy sooner (nor any questions on pregnancy attempt, duration, or lifetime experiences of infertility). Existing research on pregnancy desires and intentions in sub-Saharan Africa has found characteristics consistently associated with unintended compared to intended pregnancy, for instance education, wealth, age, marital status, parity, and residence [26, 27, 42], but we have limited knowledge of the ways in which women who have pregnancies later than desired compare to those who have a child at a preferred time.

The purpose of this study is to examine desired pregnancy timing in Uganda. While fertility in Uganda has declined in recent years from approximately 6.8 in 2000 to 4.6 in 2022 [43], fertility desires remain high. The mean ideal number of children among all women was 4.8 in 2016, with approximately 23% of women reporting wanting to become pregnant within 2 years [44]. At the same time, unintended pregnancy is high, with approximately 46% of women who gave birth in the past five years stating their most recent pregnancy was either mistimed or unwanted [45]. High wanted fertility and unintended pregnancy rates, however, co-exist with high unrealized fertility; a recent study estimated that approximately 46% of women in Uganda at the end of their reproductive years (aged 44–48) had unrealized fertility, having reported a higher ideal number of children than their current number of children [34]. The primary objective of our study is to determine the full distribution of desired pregnancy timing—disaggregating those who had a later-than-desired pregnancy from those who wanted it then—among recent births and current pregnancies and evaluate sociodemographic and reproductive factors associated with having a later-than-desired pregnancy. Our secondary objective is to examine how these factors differ when comparing those who experienced a pregnancy later than desired to those who experienced their pregnancy when they desired, as this is the group that is most often combined in existing literature.

## Methods

### Data source

We use data from the Performance Monitoring for Action (PMA) Uganda 2022 survey [46]. In 2020, PMA utilized multi-stage cluster sampling with urban/rural stratification and probability proportional to size selection to identify 141 enumeration areas (EAs). After listing all households in each EA, 35 households were randomly selected for interview. All women aged 15–49 who were either regular members of the household or who slept in the household the night before were eligible for interview and, if they provided informed consent, were enrolled in a panel study (Phase 1). Experienced and trained enumerators explained study procedures and administered informed consent. Written consent was provided by women aged 18–49 or emancipated minors 15–17 years, and written parental consent and individual assent was obtained from non-emancipated girls aged 15–17. In 2021 (Phase 2) and 2022 (Phase 3), interviewers returned to all Phase 1 households to reinterview women who had consented for follow-up, also adding 19 additional cluster in 2021 via the same sampling strategy used in 2020. If panel women were unable to be relocated, they were dropped from the panel. If a new woman joined a panel household or an adolescent became eligible (i.e., turned 15) they were invited to provide consent for and participate in the study. Finally, additional households were randomly selected and enrolled into the survey to replace households lost-to-follow-up or destroyed to create representative cross-sectional samples of households at each Phase. In total, 4227 (96.4% response rate) women completed the Phase 3 survey that we use in the current study.

### Measures

#### Outcome

Our primary outcome of interest is experiencing a later-than-desired pregnancy. Women who were currently pregnant or who had ever given birth were initially asked the standard PMA question to assess intendedness of their current pregnancy or most recent birth—“At the time you became pregnant, did you want to become pregnant then, did you want to wait until later, or did you not want to have any more children at all?” Women who responded that they wanted to become pregnant “then” were subsequently asked, “Did you want to become pregnant earlier”? Women who responded “yes” were considered to have had a later-than-desired pregnancy. Additionally, all currently pregnant women and pregnancies that occurred in the last two years (only one year for panel women since they were surveyed the year prior) were asked, “How many months did it take for you to become pregnant?”.

#### Explanatory

We examine several sociodemographic correlates that have previously been linked to unintended pregnancy. These include age (15–19, 20–29, 30–39, 40–49), highest level of schooling attended (never, primary, secondary, higher), marital status (currently married or cohabiting, divorced/separated or widowed, never married), wealth tertile, residence (urban, rural), parity (0, 1, 2–4, 5+ births), desire for another child (undecided, wants another child, wants no more children, reports not fertile). Additionally, we included variables assessing self-rated health (very good, good, moderate, bad, very bad), whether the respondent had ever sought care for difficulties getting pregnant, and whether the woman had correct knowledge of the fertile window, defined as being halfway between two menstrual periods. Finally, women who were currently pregnant were also asked about their emotional response to becoming pregnant, which was a five-category variable ranging from very happy to very unhappy.

### Analytic methods

We restricted the sample of women aged 15–49 to those who were either currently pregnant or who had ever given birth (n = 3318) and who answered the question on desired pregnancy timing (n = 3311). We first used descriptive statistics to examine the charactistics of women in the analytic sample. We then compared the characteristics of women across desired pregnancy timing categories (later-than-desired, desired time, earlier-than-desired, undesired). Statistically significant differences were identified using design-based F-statistics. Next, we used multivariable logistic regression to compare those who reported wanting their pregnancy earlier (i.e., had later-than-desired pregnancy) versus then (i.e., at desired time)—the group of women who are most often combined in other studies—accounting for all variables described above, except for happiness with the current pregnancy as it was only asked of currently pregnant women. Given the outcome is common, our odds ratios should not be interpreted as risk ratios as they would overestimate the risk. Finally, we describe the number of months that women reported they had tried to become pregnant, overall and by fertility intention category. This question was only asked of women who were currently pregnant, panel women who had given birth in the last year, and newly consented Phase 3 cross-sectional women who had given birth in the last two years. To account for multistage sampling, differential probability of selection, and non-response, all analyses applied survey design weights and accounted for clustering within EAs.

## Results

**Fig. 1:**
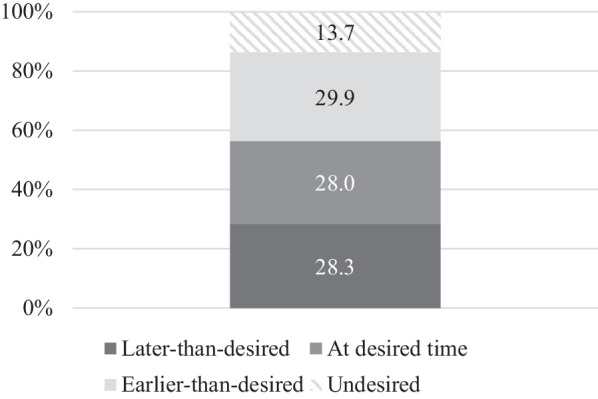
Desired timing of current or most recent pregnancy among women aged 15–49 in Uganda who are currently pregnant or have ever given birth (N = 3311)

Our total analytic sample included 3311 reproductive-aged women who were currently pregnant or had ever given birth. A similar percentage of women had a later-than-desired pregnancy (28.3%), a pregnancy at the desired time (28.0%), and an earlier-than-desired pregnancy (29.9%), while 13.7% had an undesired pregnancy (Fig. 1). The sample was predominantly aged 20–29 (39.6%) and the majority had attended primary school (57.6%), were currently married or cohabiting (75.3%), and resided in a rural area (71.2%) (Table 1). One-in-three women had 5 or more children and 63.7% wanted to have a/another child, while more than 40% of those who were currently pregnant reported they were very happy when they found out. Nearly half (48.1%) indicated they considered their health “good” and only 5.8% had ever sought care for difficulties getting pregnant.

**Table 1:** Characteristics of women aged 15–49 in Uganda who are currently pregnant or have ever given birth, overall and by desired pregnancy timing and background characteristics, 2021

Characteristic	Total		Desired pregnancy timing							
			Later-than-desired		Desired time		Earlier-than-desired		Undesired	
	N	%	N	%	N	%	N	%	N	%
Age										
15–19	241	7.6	**52**	**6.5**	**49**	**5.1**	**105**	**11.2**	**35**	**6.8**
20–29	1309	39.6	**349**	**43.2**	**402**	**39.0**	**455**	**43.1**	**103**	**25.5**
30–39	1116	34.2	**295**	**34.5**	**359**	**35.4**	**326**	**34.0**	**136**	**31.6**
40–49	645	18.7	**145**	**15.8**	**215**	**20.6**	**132**	**11.6**	**153**	**36.1**
Education										
Never	219	5.0	**57**	**3.4**	**89**	**6.6**	**42**	**4.0**	**31**	**7.2**
Primary	2012	57.6	**452**	**49.2**	**604**	**58.6**	**671**	**61.6**	**285**	**64.1**
Secondary	857	28.8	**250**	**33.7**	**262**	**26.4**	**249**	**27.8**	**96**	**25.4**
Higher	222	8.6	**82**	**13.7**	**69**	**8.4**	**56**	**6.6**	**15**	**3.2**
Marital status										
Currently married/cohabiting	2475	75.3	**678**	**80.3**	**771**	**75.9**	**735**	**73.7**	**291**	**67.5**
Divorced or separated/widowed	678	19.5	**148**	**17.5**	**228**	**21.5**	**202**	**17.3**	**100**	**24.2**
Never married	157	5.2	**15**	**2.3**	**25**	**2.6**	**81**	**9.0**	**36**	**8.3**
Wealth tertile										
Poorest	1278	33.2	**288**	**24.5**	**373**	**34.6**	**441**	**39.0**	**176**	**35.3**
Middle wealthiest	1155	34.1	**291**	**34.3**	**359**	**32.9**	**342**	**32.6**	**163**	**39.2**
Wealthiest	878	32.7	**262**	**41.2**	**293**	**32.4**	**235**	**28.3**	**88**	**25.5**
Residence										
Rural	2212	71.2	484	65.7	700	71.5	710	73.8	318	76.4
Urban	1099	28.8	357	34.3	325	28.5	308	26.2	109	23.6
Parity										
0	89	2.9	**37**	**5.0**	**21**	**2.0**	**25**	**2.4**	**6**	**1.5**
1	696	21.7	**174**	**23.4**	**199**	**19.3**	**238**	**23.8**	**85**	**18.5**
2–4	1381	42.0	**396**	**47.0**	**488**	**48.5**	**414**	**41.2**	**83**	**20.5**
5+	1145	33.4	**234**	**24.7**	**317**	**30.1**	**341**	**32.6**	**253**	**59.6**
Desire for a/another child										
Undecided/do not know	101	3.2	**23**	**3.6**	**24**	**2.4**	**36**	**3.2**	**18**	**4.2**
Have a/another child	2106	63.7	**602**	**72.3**	**676**	**66.0**	**680**	**66.9**	**148**	**34.5**
No more	1043	31.5	**197**	**22.1**	**308**	**30.4**	**288**	**28.9**	**250**	**58.9**
Says can’t get pregnant	51	1.5	**16**	**2.0**	**14**	**1.1**	**10**	**1.0**	**11**	**2.4**
Emotional response to pregnancy (among currently pregnant)										
Very happy	148	42.1	**84**	**70.8**	**59**	**62.0**	**5**	**2.8**	**0**	**0.0**
Sort of happy	59	16.7	**19**	**18.1**	**14**	**13.5**	**24**	**20.0**	**2**	**7.7**
Mixed happy and unhappy	36	8.3	**4**	**2.7**	**8**	**8.0**	**22**	**16.0**	**2**	**6.4**
Sort of unhappy	47	11.5	**1**	**0.8**	**7**	**6.8**	**34**	**28.2**	**5**	**12.2**
Very unhappy	94	21.4	**14**	**7.6**	**8**	**9.6**	**46**	**33.0**	**26**	**73.7**
General health										
Very good	618	17.2	**158**	**16.0**	**234**	**23.5**	**171**	**15.1**	**55**	**11.8**
Good	1560	48.1	**397**	**51.9**	**496**	**46.2**	**487**	**48.7**	**180**	**43.0**
Moderate	898	28.0	**235**	**26.7**	**226**	**24.0**	**291**	**30.0**	**146**	**34.8**
Bad	207	5.6	**41**	**4.2**	**62**	**5.6**	**64**	**5.4**	**40**	**8.9**
Very bad	28	1.0	**10**	**1.3**	**7**	**0.6**	**5**	**0.9**	**6**	**1.5**
Ever sought care for difficulties getting pregnant										
No	3108	94.2	**759**	**90.9**	**971**	**95.3**	**968**	**95.2**	**410**	**96.7**
Yes	194	5.8	**82**	**9.1**	**50**	**4.7**	**47**	**4.8**	**15**	**3.3**
Correct knowledge of fertile window										
No	2662	81.2	682	82.8	817	79.3	818	80.8	345	82.9
Yes	649	18.8	159	17.2	208	20.7	200	19.2	82	17.1
Total	3311	100.0	841	100.0	1025	100.0	1018	100.0	427	100.0

Bolding indicates statistically significantly different at the p < 0.05 level from design-based F-test, italics indicate significant at p < 0.10 level; percentages are weighted, Ns are unweighted

Nearly all sociodemographic and reproductive characteristics were associated with the desired pregnancy timing of women’s current or most recent pregnancy (Table 1). Women who had a later-than-desired pregnancy were on average 30.5 years old, while those who had a pregnacy at the desired time (31.8) or an undesired pregnancy (34.4) were older and those who had an earlier-than-desired pregnancy were younger (29.1) (results not shown). Those with secondary or higher education and the wealthiest women were more likely to report having a later-than-desired pregnancy. Women with fewer children and those who wanted to have a/another child were also more likely to report a later-than-desired pregnancy. The largest observed differences across desired pregnancy timing were seen in relation to emotional response to the pregnancy, with those who had a later-than-desired pregnancy much more likely to report they were very happy. Those who had a later-than-desired pregnancy, earlier-than-deisred pregnancy, or undesired pregnancy were less likely to report being in very good health, while those who had a later-than-desired pregnancy were most likely to have sought care for difficulties getting pregnant. There were no differences by residence and correct knowledge of the fertile window.

Table 2 shows adjusted odds ratios of having a later-than-desired pregnancy relative to women who wanted to become pregnant then. Having greater education was independently associated with increased odds of having a later-than-desired pregnancy, with attending higher education specifically associated with 2.41 [95% confidence interval (CI) 1.13–5.13] times the odds of having a later-than-desired pregnancy. Those with no children had nearly twice the odds [adjusted odds ratio (aOR) 1.98, 95% CI 0.99–3.95] of experiencing a later-than-desired pregnancy compared to those with one child while women who had sought care for difficulties getting pregnant had 2.12 (95% CI 1.30–3.46) times the odds of a later-than-desired pregnancy compared to those who had never sought care. Lastly, women with self-reported good or moderate health both had just over 1.70 (95% CI 1.12–2.71 and 1.09–2.86, respectively) times the odds of experiencing a later-than-desired pregnancy compared to those with very good health while those with self-reported very bad health had more than four (aOR 4.32, 95% CI 1.15–16.26) times the odds of having a later-than-desired pregnancy.

**Table 2:** Adjusted odds ratios of having a later-than-desired pregnancy compared to having a pregnancy when desired among women aged 15–49 in Uganda who are currently pregnant or have ever given birth (n = 1854), 2021

	aOR	95% CI	
Age (ref. 15–19)			
20–29	0.79	0.45	1.40
30–39	0.76	0.40	1.42
40–49	0.77	0.36	1.65
Education (ref. never)			
Primary	1.33	0.73	2.40
Secondary	*1.75*	*0.96*	*3.17*
Higher	**2.41**	**1.13**	**5.13**
Marital status (ref. currently married/cohabiting)			
Divorced or separated/widowed	0.83	0.55	1.25
Never married	0.54	0.24	1.24
Wealth tertile (ref. poorest)			
Middle wealthiest	1.37	0.90	2.10
Wealthiest	1.42	0.72	2.80
Residence (ref. rural)			
Urban	1.10	0.53	2.26
Parity (ref. 1)			
0	*1.98*	*0.99*	*3.95*
2–4	0.83	0.53	1.31
5+	0.91	0.53	1.56
Desire for a/another child (ref. undecided/do not know)			
Have a/another child	0.67	0.29	1.51
No more	*0.50*	*0.23*	*1.10*
Says can’t get pregnant	1.24	0.37	4.15
General health (ref. very good)			
Good	**1.74**	**1.12**	**2.71**
Moderate	**1.77**	**1.09**	**2.86**
Bad	1.31	0.69	2.47
Very bad	**4.32**	**1.15**	**16.26**
Sought care for difficulties getting pregnant (reg. No)			
Yes	**2.12**	**1.30**	**3.46**
Correct knowledge of fertile window (ref. No)			
Yes	0.76	0.52	1.11

Bolding indicates statistically significantly different at the p < 0.05 level, italics indicate significant at p < 0.10 level

Among the subsample of 907 women who were currently pregnant or who had a recent pregnancy, we did not observe significant differences in the distribution of the number of months women had been trying to become pregnant by desired pregnancy timing (Table 3). However, 6.3% of those who had a later-than-desired pregnancy had been trying two or more years, which was the highest among the desired pregnancy timing groups. Additionally, those who had a later-than-desired pregnacy had the highest proportion of people who reported trying less than 6 months (63.2%).

**Table 3:** Number of months had tried to get pregnant among women aged 15–49 in Uganda who are currently pregnant or recently gave birth

Number of months tried to get pregnant	Total		Desired pregnancy timing							
			Later-than-desired		Desired time		Earlier-than-desired		Undesired	
	N	%	N	%	N	%	N	%	N	%
< 6	515	59.5	158	63.2	140	60.8	169	57.1	48	51.0
6–11	270	26.2	82	24.6	75	28.3	85	24.5	28	32.9
12–23	72	9.3	17	5.9	12	5.7	34	14.4	9	12.5
24+	50	5.0	22	6.3	13	5.3	12	4.0	3	3.6
Total	907	100.0	279	100.0	240	100.0	300	100.0	88	100.0

Percentages are weighted, Ns are unweighted

## Discussion

This study reveals a substantial proportion of women in Uganda who had a later-than-desired pregnancy (28%). This group of women constituted half of those who identified as wanting their pregnancy at that time in the absence of an option to indicate they wanted it earlier. Thus, there is a significant portion of women whose actual desired pregnancy timing is not captured in reproductive health surveys implemented in the Global South. Though we have no comparable research from other sub-Saharan Africa or low-resource settings, our finding is much larger than the percent who reported having a later-than-desired pregnancy in the United States [16, 31], suggesting this is perhaps a more significant issue in this context.

Desired pregnancy timing—including having a later-than-desired pregnancy—was highly related to sociodemographic and reproductive characteristics, including education, age, marital status, wealth, and parity, which is consistent with existing literature on factors associated with unintended pregnancy in sub-Saharan Africa [26, 27, 42]. In addition to including having a later-than-desired pregnancy, we extend this prior work by also examining the relationship between desired pregnancy timing and emotional response to the pregnancy as well as perceptions of general health and whether one sought care for difficulties getting pregnancy, all of which were highly related to desired pregnancy timing in our study. Specifically in comparison to those who wanted the pregnancy then, those who had a later-than-desired pregnancy were more likely to have secondary or higher education, more likely to be nulliparous, more likely to report less than very good health, and more likely to have sought care for difficulties getting pregnant (though care seeking was very low even among this group).

While these findings highlight an important gap in our understanding of desired pregnancy timing and intentions, these data do not reveal the reason for the delayed fertility. Our results suggest multiple factors contribute to delayed fertility, including both potential fecundity related issues and life circumstances. People who had a later-than-desired pregnancy in our study were more likely to report having less than very good health. This perception could arise as a result of having difficulty conceiving or, conversely, could signal an underlying health issue that may affect fecundity. Little research has explored the relationship between fecundity and self-rated health in sub-Saharan Africa. Rao and colleagues found no association between self-reported infertility and self-rated health in Malawi [47], however, theirs is the only study we identified that explored this specifically, underscoring the dearth of research in this area. Our results also indicate some women may have had a later-than-desired pregnancy due to life circumstances. Women with more schooling (and who are wealthier, which is correlated) were more likely to report having a later-than-desired pregnancy, perhaps signaling that educational and professional aspirations may compete with achievement of one’s reproductive goals. Evidence suggests Uganda’s elimination of primary school fees resulted in women staying in school longer, leading them to delay marriage and their first birth, supporting this potential explanation [48]. This is an issue of subjective infertility [49, 50], less so an issue of biomedical infertility/delayed conception, though given fecundity decreases with age, they may be interrelated.

These two potential sources of delay—biomedical and life circumstances—are not mutually exclusive but can both contribute to one having a later-than-desired pregnancy. In the absence of information about the reason for the delay, duration of attempt conflates time-to-pregnancy for those with fertility issues and those without fertility issues who were simply delayed in initiating their pregnancy attempt. The lack of an observed relationship between desired pregnancy timing and number of months they had been trying to conceive in our study is, thus, perhaps unsurprising. Further work is needed to understand the reasons contributing to one’s later-than-desired pregnancy and how delayed the pregnancy was from some optimal timing.

Another noteworthy finding was the non-trivial (7.6%) portion of women who had a later-than-desired pregnancy who indicated they were very *un*happy with the pregnancy. This highlights the complex nature of fertility desires and intentions, which can be influenced by many time-varying factors, including relationship stability, perceived economic and health security, and other personal life circumstances [51–56]. Those who experience difficulties getting pregnant may also adapt their pregnancy desires to resolve cognitive dissonance of unachieved fertility [57, 58]. Thus, we can’t assume all pregnancies that result following a period of infertility are intended [59]; people who experience infertility can go on to have later-than-desired pregnancies that are in fact unintended [60]. This nuance is generally not captured in the existant literature on desired pregnancy timing, particularly in sub-Saharan Africa.

This study has a number of limitations. The primary limitation is that our data do not allow us to determine the reason for the delay, specifically whether it is potentially a biological impairment contributing to delayed conception or a delay due to social, economic, or other circumstancial factors [49, 50]. Our results and associations conflate these two groups, which may in fact be distinct. Among those experiencing a biological impairment, the delay could be related to recent hormonal contraceptive use as some hormonal methods are shown to be associated with delayed return to fecundity [61, 62], however, we did not have the necessary data to explore this potential explanation. Additionally, we do not know specifically when the women in our study preferred to have a child as we only asked how long they had been trying, which may be inadequate to assess the extent of mistiming for those not experiencing a biological impairment. Our data also may suffer from ex post facto rationalization or recall bias as reports of desired pregnancy timing were retrospective [63]. Lastly, our data do not include women who were not able to get pregnant at all, thus these results do not capture the full extent of people who desired a pregnancy earlier, and the associations may be different or stronger when accounting for this population.

Despite these limitations our study has a number of strengths. To our knowledge this is the first study to assess this measure in sub-Saharan Africa. We used a large, nationally representative sample of reproductive-aged women and were able to examine the relationship between desired pregnancy timing and many socioeconomic and reproductive characteristics, as well as novel measures on self-reported health, whether sought care for issues getting pregnant, and emotional reaction to the pregnancy. Further research is needed to understand the factors contribuing to having a later-than-desired pregnancy, as well as the myriad potential impacts of this experience.

## Conclusions

The global family planning community is moving towards embracing a reproductive justice framework and is working towards the development of new indicators [64, 65], but much work remains. Disaggregating those who wanted a pregnancy earlier and measuring it—as well as the extent of infertility—are essential if we seek to make progress towards supporting women and couples in achieving their reproductive goals, not just preventing pregnancies through contraceptive use. Research on desired pregnancy timing in sub-Saharan Africa should be expanded to capture later-than-desired pregnancies, a population which is invisible in existing data. This work has public health implications due to commonalities in the factors associated with mistimed and unintended pregnancies and their link to poorer health and potentially poorer pregnancy outcomes.

## Data Availability

This study used the PMA Uganda Phase 3 household and female survey data, which are publicly available at https://doi.org/10.34976/6mkm-a674.

## Abbreviations

CI: Confidence interval
EA: Enumeration area
PMA: Performance Monitoring for Action
